# Radar Signal Modulation Recognition Based on Sep-ResNet

**DOI:** 10.3390/s21227474

**Published:** 2021-11-10

**Authors:** Yongjiang Mao, Wenjuan Ren, Zhanpeng Yang

**Affiliations:** 1 Key Laboratory of Network Information System Technology, Institute of Electronics, Chinese Academy of Sciences, Beijing 100190, China; myj@whu.edu.cn (Y.M.); zhanpengyang@mail.ie.ac.cn (Z.Y.); 2 Aerospace Information Research Institute, Chinese Academy of Sciences, Beijing 100190, China; 3 University of Chinese Academy of Sciences, Beijing 100049, China

**Keywords:** radar modulation signal, time–frequency analysis, complex Morlet wavelet, image enhancement, channel-separable ResNet

## Abstract

With the development of signal processing technology and the use of new radar systems, signal aliasing and electronic interference have occurred in space. The electromagnetic signals have become extremely complicated in their current applications in space, causing difficult problems in terms of accurately identifying radar-modulated signals in low signal-to-noise ratio (SNR) environments. To address this problem, in this paper, we propose an intelligent recognition method that combines time–frequency (T–F) analysis and a deep neural network to identify radar modulation signals. The T–F analysis of the complex Morlet wavelet transform (CMWT) method is used to extract the characteristics of signals and obtain the T–F images. Adaptive filtering and morphological processing are used in T–F image enhancement to reduce the interference of noise on signal characteristics. A deep neural network with the channel-separable ResNet (Sep-ResNet) is used to classify enhanced T–F images. The proposed method completes high-accuracy intelligent recognition of radar-modulated signals in a low-SNR environment. When the SNR is −10 dB, the probability of successful recognition (PSR) is 93.44%.

## 1. Introduction

Radar modulation signal (RMS) recognition is the basis of radar electronic countermeasures and electronic jamming, and is a necessary problem in electronic warfare [1]. With the use of various multi-band and full-coverage communication equipment, electronic interference and signal aliasing have appeared in space. This makes the electromagnetic environment more complicated [2,3], which brings difficulties to the recognition of RMS in low-SNR environments. Because RMS has good performance in complex spaces, it is important to improve the probability of the successful recognition of RMS in low-SNR environments.

For the traditional recognition methods of RMS, those methods based on signal characteristic parameter matching proposed by [4] and the judgment method based on the expert system proposed by [5] are used to identify RMS. Both of them are disturbed by human factors and are not stable. In [6], the principal component analysis (PCA) was used to extract radar signal features. In [7], the support vector machine (SVM) and T–F distribution images of signals were used to identify RMS. These methods, based on traditional machine learning, tend to select the characteristics of RMS manually. The methods require much a priori knowledge and struggle to meet the recognition requirements for the new radar systems and the variety of modulated signals [8]. With the development of artificial intelligence, deep learning has also been applied to the recognition of radar signals. Classification and recognition based on deep learning have many advantages. Without human assumptions and intervention about the features to be extracted, the deep neural network can effectively learn the features of the signals [9]. Deep learning can better resist the interference of noise in the extraction of signal features, thereby improving the generalization ability and accuracy in the identification of RMS [10]. In [11], the input of the classification network is a one-dimensional RMS sequence based on the time domain, frequency domain, and autocorrelation domain. By combining a convolutional neural network (CNN), a long short-term memory network (LSTM), and a deep neural network (DNN), the recognition of RMS was completed. However, this method completed the signal recognition on a one-dimensional basis. The method had no denoising processing in the case of one-dimensional signal sequences. The result was not ideal in a low-SNR environment of ≤−10 dB. In [12], the improved AlexNet and Choi–Williams T–F distribution is used to complete the recognition of RMS based on two-dimensional images. This method did not denoise the T–F images, and the classification network was designed simply, resulting in weak anti-noise ability. This method cannot extract the features of T–F images well, and the PSR is low. In [13], the Cohen T–F transform method was used to convert the RMS to obtain T–F images, and the surface features of the image were extracted by CNN, and then the recurrent neural network was used to classify the T–F images of RMS. Because the images obtained by the Cohen transform had strong cross-terms, the characteristics of the signal experienced interference by the cross-terms, and the network could not extract the T–F image features well in low-SNR environments, resulting the PSR of the radar signal not being ideal when the SNR was −8 dB.

In this paper, in response to the difficulty in recognizing the RMS in low-SNR environments, we propose a novel method that combines the CMWT and the Sep-ResNet to accurately identify the RMS in a low-SNR environment of −13 dB. Through the enhancement T–F images by CMWT and the classification network of Sep-ResNet, the method in this paper has a strong anti-noise interference ability. This method can identify seven types of RMS, including normal signal (NS), linear frequency modulation (LFM), non-linear frequency modulation (NLFM), two-frequency shift keying (2FSK), two-phase shift keying (2PSK), four-frequency shift keying (4FSK) and four-phase shift keying (4PSK). The overall PSR of our method for seven types of RMS could reach 96.57% from 8 dB to −13 dB. In the case of SNR ≥ 2 dB, the PSR was 100%. In the case of low-SNR environments of −10 dB and −13 dB, the PSR was 93.44% and 88.24%, respectively.

Our major contributions are summarized as follows:

- The CMWT was introduced into the T–F analysis, which made it possible to avoid the interference of the T–F distribution cross-terms in the signal characteristics, and the T–F images had high T–F resolutions;
- The T–F images were denoised and enhanced through adaptive filtering and morphological methods. Effective morphological structural elements were designed to filter out noise on the T–F images and reduce the interference of noise in signal characteristics;
- By improving the residual unit structure, named Sep-ResNet, and multiple receptive fields for extracting features, as well as fusing multi-channel feature maps, the PSR was improved 2.51% in a low-SNR environment of −13 dB.

The remainder of this paper is organized as follows. Section 2 introduces the related work in the field of radar-modulation signal recognition. Section 3 introduces the recognition system framework and the proposed method, including the T–F analysis of CMWT, the T–F image-enhancing algorithm, and the improved classification network of Sep-ResNet. Section 4 shows the experimental data and results, and discusses the effectiveness of our method. Finally, Section 5 includes the conclusion of the whole work.

## 2. Related Work

In the past, many scholars have devoted themselves to exploring the automatic recognition system of RMS in applications. They have proposed several practicable approaches, making the system more intelligent, more robust, and less artificial. These achievements have pushed forward the development of the field of RMS recognition.

The recognition of RMS includes the extraction and classification of characteristics. In [7], the T–F analysis was used to extract signal characteristics. The SVM and auto encoder were used to classify the signal. This method introduced a slack variable to consider a non-linearly separable problem to find the best hyperplane so that the classification result had widths of the maximum margin. The method solved the problem of high-dimensional classification by selecting a suitable kernel function. The method identified RMS successfully, and the PSR was 82% in an SNR environment of −6 dB. The authors of [11] proposed a network combining CNN, LSTM, and DNN. They successfully identified six types of RMS when the SNR was from −14 to 20 dB. This method extracted the characteristics of the signal as the original one-dimensional sequence in the time domain, the fast Fourier transform sequence in the frequency domain, and the result of signal autocorrelation in the autocorrelation domain. A CNN was used to extract the surface features of the signal in different domains, and the features extracted by the CNN were used as the input of the LSTM, and the DNN was used to classify the characteristics of the signal. The length of the signal sequence extracted by this method needed to be set in advance, and the sequence length was different in the time domain, frequency domain, and autocorrelation domain. Under the preset optimal sequence length, the PSR was about 90% when the SNR was −6 dB. This method required preprocessing to obtain the optimal sequence length of a signal for a specific domain, which had limitations. Moreover, the features of one-dimensional sequence were not as rich as that of two-dimensional T–F images, and the recognition accuracy was not as high as that obtained in [14]. When signal features were extracted by the one-dimensional sequence, some feature parameters needed to be manually selected for the data. The T–F analysis method can overcome the shortcomings of the Fourier transform and reflect the signal characteristics in the two-dimensional space of T–F. By obtaining the T–F images, the order of appearance of each frequency component can be well distinguished. The T–F analysis method can adequately extract the characteristics of non-stationary signals, such as RMS. In [12], the Choi–Williams distribution (CWD) was a method of T–F analysis that was used to extract the features of a modulated signal x(t). The expression is as follows:

(1) $$C_{x}\left(t,\omega \right)=\frac{1}{4\pi^{2}}{∭}_{\infty }^{\;}\phi \left(\tau ,v\right)e^{-j\left(vt+\omega \tau \right)}x\left(u+\frac{\tau }{2}\right)x^{*}\left(u-\frac{\tau }{2}\right)e^{jvu}dvd\tau du$$

where t and ω are the time and frequency coordinates, respectively. $x^{*}\left(t\right)$ is the conjugate expression of x(t). ϕ(τ,v) and σ are the kernel function and filter bandwidth, respectively. In [12], the kernel function was a Gaussian kernel $\phi \left(\tau ,v\right)=exp\left[-\frac{{\left(\tau v\right)}^{2}}{\sigma }\right]$ and σ=1. The larger σ, the better aggregation of signal on the T–F images. However, a large σ will bring more serious cross-terms by x(t) times $x^{*}\left(t\right)$. Cross-terms will reduce the quality of T–F images. In the improved AlexNet, the dropout is added to reduce overfitting, the size of the convolution kernel is modified, the receptive field of convolution is increased, and the fully connected layer is reduced to decrease the weight parameters. When the SNR is −2 dB, the recognition rate can reach 80%. In [15], the signal characteristics were extracted through the improved phase difference short-time Fourier transform (STFT). The STFT of the signal is expressed as follows:

(2) $$STFT\left(t,f\right)=\int_{-\infty }^{+\infty }x\left(\tau \right)w\left(\tau -t\right)exp\left(-j2\pi f\tau \right)d\tau \;$$

where w(t) and x(t) are fixed-width window functions and signals to be analyzed, respectively. Through the continuous sliding of the w(t) window, the Fourier transform was performed in the window to extract the signal characteristics. It reduced the influence of noise by increasing the order of the phase difference. However, an increase in the phase difference order would increase the complexity of the algorithm. In addition, the STFT used a fixed-width window function to extract signal characteristics, resulting in low T–F resolution. When the SNR was −6 dB, the PSR of recognition result was 93%. In [14], by improving the kernel function of CWD, the original Gaussian kernel function was changed to $\phi \left(\tau ,v\right)=exp\left(-\frac{\alpha \tau^{2}+\beta v^{2}}{\sigma }\right)$, which solved the problem of ϕ(τ,v)=0 when τ=0 or v=0. To a certain extent, it was able to reduce the interference of the cross-terms in the T–F distribution images. Furthermore, the T–F images were denoised by 2D-Wiener filtering. This method improved the PSR to 96% when the SNR was −6 dB, and the PSR was found to be 78% when the SNR was −8 dB. Due to the existence of cross-terms, this method does not perform well in low-SNR environments. In [16], the wavelet transform T–F analysis method was used to extract the characteristics of the signal. The wavelet transform represents an improvement upon STFT; the signal to be analyzed is decomposed into a series of superpositions of wavelet functions. The wavelet functions are obtained from the mother wavelet through translation and scaling transformation. The mother wavelet is stretched at low frequencies and compressed at high frequencies, and has the characteristics of multi-scale refinement. In order to restore the characteristics of the signal on the time and frequency scale, the wavelet transform method continually approximates the signal that is to be analyzed.

A large number of RMS recognition systems have been designed, but the identification of a variety of RMS with high accuracy in low-SNR environments of ≤−10 dB remains a challenging problem.

## 3. System Framework and Method

**Figure 1:**
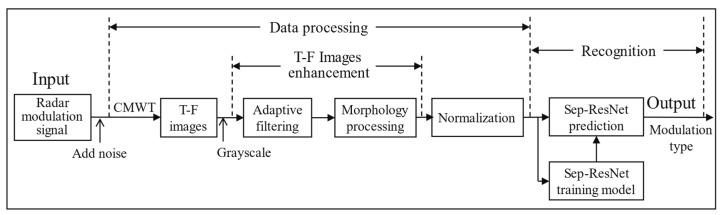
RMS recognition system framework.

This paper designs an intelligent method to identify RMS with high accuracy in low-SNR environments. The first step is to transform the RMS into two-dimensional T–F images using CMWT. In the second step, the T–F images are grayed out, and the T–F images are denoised and enhanced through adaptive filtering and morphological processing to reduce the interference of noise on signal characteristics. In the last step, the enhanced T–F images are fed into the Sep-ResNet to train the model, and the trained model is used to accurately predict the type of RMS. The system framework is shown in Figure 1.

The received RMS model is as follows:

(3) x(t)=s(t)+n(t)  

where x(t) represents the received RMS, and t is the time variable; s(t) and n(t) represent the transmitted RMS and the received noise, respectively. The transmitted RMS is as follows:

(4) $$s\left(t\right)=Arect\left(\frac{t}{T}\right)e^{-j\left(2\pi f_{c}t+\phi \left(t\right)+\phi_{0}\right)}\;$$

(5) $$rect\left(\frac{t}{T}\right)=\{\begin{array}{c} 1,\left|t/T\right|\leq 1/2\\ 0,\left|t/T\right|\geq 1/2 \end{array}$$

where A represents the signal amplitude, and A=1 during simulation. The rect(•) is the rectangular threshold function as shown in Equation (5), T is the pulse width of the signal, $f_{c}$ is the signal carrier frequency, $\phi_{0}$ is the initial phase of the signal, and ϕ(t) is the different phase function, which determines the different signal modulation modes.

To simulate the received noise in the real channel of signals, Gaussian noise, white noise, narrow-band Gaussian noise, and carrier frequency random disturbance are added [17]. Gaussian noise and white noise are some of the most common noises in actual channels. The probability density function of Gaussian noise obeys a normal distribution, with a mean value of 0 and a variance of 1. The power spectral density of white noise is a constant over the entire bandwidth and obeys a uniform distribution, $S\left(\varpi \right)=N_{0}/2,\varpi \in \left(-\infty ,+\infty \right)$. When adding white noise, it only needs to be added within the actual signal bandwidth. Often, in a real radar communication system, a band-pass filter of the target signal bandwidth is added at the receiving end. Because the communication frequency of the radar signal is high, much larger than the bandwidth of the band-pass filter, the generation of Gaussian narrow-band noise occurs. Narrow-band Gaussian noise obeys the Rayleigh distribution on the random envelope and obeys the uniform distribution on the phase. It is generally a stationary random process. Its mathematical model is $\bar{n\left(t\right)}=n_{c}\left(t\right)cos\varpi_{c}t-n_{s}\left(t\right)sin\varpi_{c}t$, where $\bar{n\left(t\right)}$ represents the average power of narrow-band Gaussian noise, $n_{c}\left(t\right)$ is the co-directional component of $\bar{n\left(t\right)}$, and $n_{s}\left(t\right)$ is the orthogonal component of $\bar{n\left(t\right)}$. The mean value of each component is $E\left[\bar{n\left(t\right)}]=E[n_{c}\left(t\right)]=E[n_{s}\left(t\right)\right]=0$, and the variance is $\sigma^{2}{}_{n}$=1. The jitter component of the carrier frequency is set to a random number in the range of 0 to 0.05 multiplied by the carrier frequency to simulate the error of the actual transmission carrier frequency and the interference received during transmission.

The T–F analysis method can transform one-dimensional signal sequences into two-dimensional T–F images to obtain RMS characteristics. Through the image enhancement method, the enhanced images are fed to the Sep-ResNet to complete the RMS recognition.

### 3.1. Complex Morlet Wavelet Transform

Wavelet transform (WT) is a T–F analysis method. WT decomposes the original signal into a series of superpositions of wavelet functions through mother wavelet translation and scaling transformation, which solves the problem that the fixed-width window function does not change with frequency in the STFT [15]. WT does not involve the conjugate multiplication of the signal itself, which avoids the occurrence of cross-terms in [12,15]. WT can obtain high T–F resolution. The mathematical model is as follows:

(6) $$CWT_{x}\left(a,b\right)=\langle x\left(t\right),\phi_{a,b}\left(t\right)\rangle =\int_{-\infty }^{+\infty }x\left(t\right)\phi^{*}{}_{a,b}\left(t\right)dt\;=\frac{1}{\sqrt{a}}\int_{-\infty }^{+\infty }x\left(t\right)\phi^{*}\left(\frac{t-b}{a}\right)dt\;$$

where x(t) is the signal to be analyzed, and $\phi^{*}{}_{a,b}\left(t\right)$ is a series of wavelet functions after the mother wavelet is translated and stretched. The transformation of the mother wavelet is as follows:

(7) $$\phi^{*}{}_{a,b}\left(t\right)=\frac{1}{\sqrt{a}}\phi \left(\frac{t-b}{a}\right)\;$$

where a is the scale expansion factor and a≠0. When a increases, $\phi_{a,b}\left(t\right)$ will widen and the amplitude will decrease, showing that the wavelet is caused by the compression of the amplitude and the stretching of the width, corresponding to the analysis of low-frequency signals. When a decreases, the wavelet becomes narrower and the amplitude increases, corresponding to the analysis of high-frequency signals. b is the time shift factor, which changes the center position of the wavelet. WT has adaptive capabilities. By selecting the appropriate mother wavelet (symmetry, orthogonality, and similarity), more detailed features can be obtained in the T–F resolution.

In the WT of our method, the complex Morlet function was chosen as the mother wavelet. CMWT is a complex exponential sinusoidal Gaussian wavelet, which has symmetry and non-orthogonality. CMWT has a good ability to extract the local characteristics of signals in the T–F domains and improves the resolution of T–F images. The complex Morlet mother wavelet model is given in Equation (8):

(8) $$\phi \left(t\right)=exp\left(\frac{-t^{2}}{2}\right)exp\left(j\omega_{0}t\right)\;$$

(9) $$\Phi \left(\omega \right)=\sqrt{2\pi }exp\left(\frac{-{\left(\omega -\omega_{0}\right)}^{2}}{2}\right)$$

**Figure 2:**
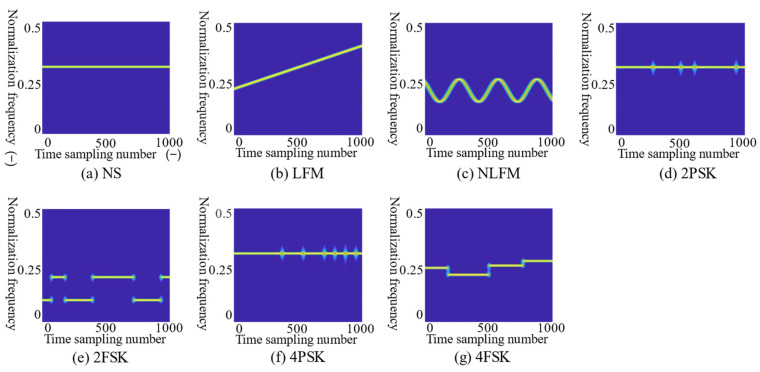
The T–F images of seven types of radar modulation signals. The images from (**a**)–(**g**) are the T–F images of NS, LFM, NLFM, 2PSK, 2FSK, 4PSK, and 4FSK without noise. The abscissa of the image is the number of sampling points. The ordinate is the normalized frequency, which is the signal frequency divide by the sampling frequency. According to the Nyquist sampling theorem, the sampling frequency must be greater than twice the signal frequency to avoid signal aliasing. The normalization frequency is between 0 and 0.5.

Equation (9) is the Fourier transform of complex Morlet wavelet, where $\omega_{0}$ represents the center frequency. The complex Morlet mother wavelet ϕ(t) is divided into two parts: the real part and the imaginary part. A series of wavelet functions $\phi^{*}{}_{a,b}\left(t\right)$ can be obtained after the translation and scaling transformation using Equation (7). Incorporating Equation (8) into Equation (6), $CWT_{x}\left(a,b\right)=CWT_{R}+jCWT_{i}$. By undertaking T–F analysis, the added imaginary part of the complex Morlet wavelet can express more changeable phase information on the original signal [18]. The complex Morlet wavelet has non-orthogonality and Gaussian adjustment, which make it possible to obtain T–F images of high time and frequency resolution through a series of variable scale wavelet functions. The CMWT avoids the interference of cross-terms in signal characteristics in low-SNR environments and improves the quality of T–F images. CMWT is suitable for the T–F analysis of RMS and can obtain clear T–F images. Figure 2 shows the T–F images of seven types of radar modulation signals of CMWT without adding noise.

**Figure 3:**
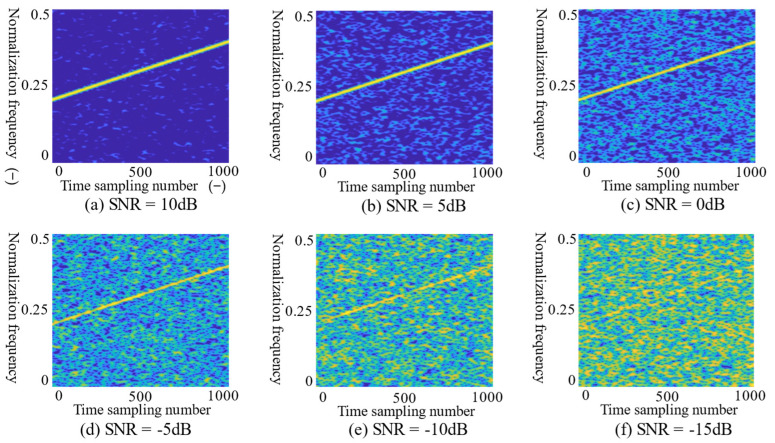
The T–F images of LFM signal in different SNR. The images from (**a**)–(**f**) are LFM T–F images under 10 dB, 5 dB, 0 dB, −5 dB, −10 dB and −15 dB noise, respectively.

In the absence of noise, the T–F images obtained by CMWT can clearly obtain the characteristics of different RMSs. The T–F images have no cross-terms interference, the signal characteristics will not be distorted, and the T–F resolution is high. Generally, the actual received radar signal will contain a lot of noise. The SNR will seriously affect the performance of the signal characteristics on the T–F images. The SNR is defined as follows:

$$SNR_{dB}=10{log}_{10}(P_{s}/P_{n})$$

(10) $$P_{s}=\frac{1}{N}\overset{N-1}{\underset{t=0}{\sum }}{\left|s\left(t\right)\right|}^{2}\;$$

$$P_{n}=\frac{1}{N}\overset{N-1}{\underset{t=0}{\sum }}{\left|n\left(t\right)\right|}^{2}\;$$

where s(t) is the modulated radar signal, n(t) is the noise signal, $P_{s}$ is the power of the signal, $P_{n}$ is the noise power, and N is the signal length. The lower SNR, the greater the noise power, and the characteristics of signal are submerged by noise on the T–F images. Figure 3 shows T–F images of the noise with different SNR added to LFM signal by CMWT.

In Figure 3, the SNR values are 10, 5, 0, −5, −10 and −15 dB, respectively. As the SNR decreases, although the characteristics of the LFM signal are still preserved, the quality of the T–F images deteriorates, and the signal characteristics are overwhelmed by noise. The difficulty of identifying the RMS will increase. Therefore, in this paper, the T–F images of the signal are properly denoised and enhanced before feeding into the CNN. T–F image enhancement can reduce the interference of noise, while better retaining the original characteristics of the signal. In addition, the enhancement algorithm improves the recognition rate of the RMS.

### 3.2. T–F Image Enhancement

**Figure 4:**
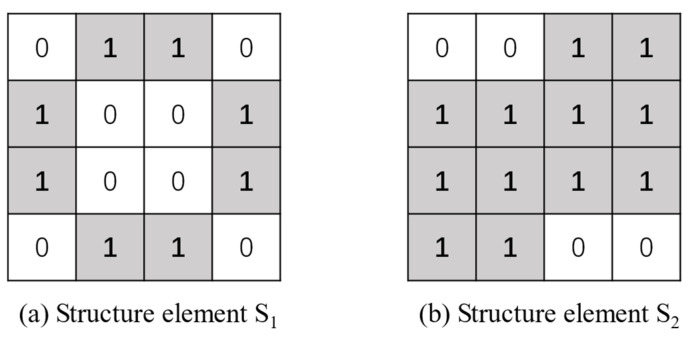
The morphological structural elements.

The T–F analysis method is usually used to extract RMS characteristics and obtain T–F images. Before recognition, it is necessary to denoise and enhance the T–F images, which includes the following steps: image cropping and gray-scale, adaptive filtering [19], morphology processing [20], and normalization. Normalization involves the down-sampling of T–F images to reshape the images into a 64 × 64-pixel form. The enhancement of the T–F images will affect the Sep-ResNet extraction features and make possible the identification of the RMS. Algorithm 1 shows the enhancement algorithm for the adaptive filtering and morphological processing of T–F images.
**Algorithm 1** Enhancement of T–F images**Input:** Grayscale images before enhancement**Adaptive Filter:****Step A:** 1: **for** origin_pixel **in** images: 2: Initialize A1, A2, window_size = 5 3: A1 = median_pixel–min_pixel, A2 = median_pixel–max_pixel 4: **if** A1 > 0 and A2 < 0: **to** Step B 5: **else**: Increase the window size 6: **if** window_size > (max_window = 13): **return** median_pixel**Step B:** 7: Initialize B1, B2 8: B1 = origin_pixel-min_pixel, B2 = origin_pixel-max_pixel 9: **if** B1>0 and B2<0: **return** origin_pixel 10: **else**: **return** median_pixel 11: **end for****Morphology Processing:** 1: Initialize the structure_element: S1, S2 2: **for** pixel **in** images: 3: the S1 Erode pixel 4: the S1 and S2 Opening Operation pixel for twice 5: the S1 Erode pixel 6: **end for****Output:** Grayscale images after enhancement
where S1 and S2 are structural elements, as shown in Figure 4:

The eroding operation is $A⊙S_{1}=\left\{z|{\left(S_{1}\right)}_{z}\subseteq A\right\}$, and S1 is the structural element for eroding. The shape of S1 is designed to be round-like, which can better eliminate round-like noise points generated on the T–F images. The dilating operation is $A⊕S_{2}=\left\{z|{\left(S_{2}\right)}_{z}\cap^{\;}A\neq \emptyset \right\}$, and S2 is used as the structural element of dilating, which can enhance the characteristics of the RMS on the T–F images. The Opening Operation is first eroding and then dilating, as in formula: $\left(A⊙S_{1}\right)⊕S_{2}$. The enhancement algorithm for T–F images is shown in Algorithm 1.

### 3.3. Classification Network of the Sep-ResNet

**Figure 5:**
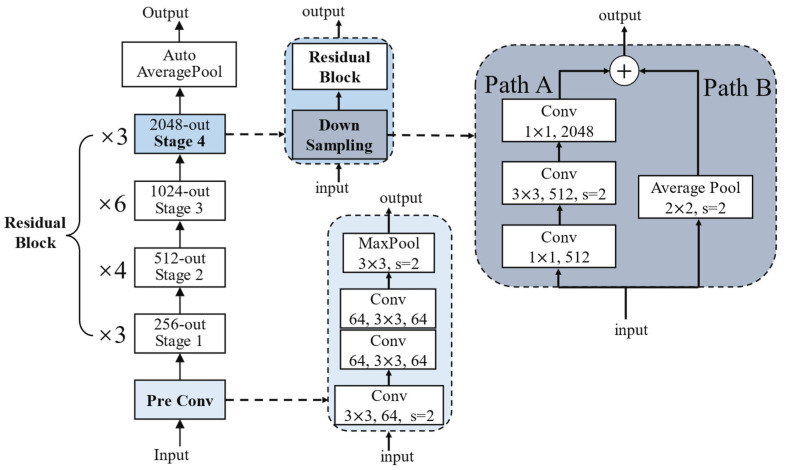
The Sep-ResNet structure developed in this paper.

After the RMS is transformed by CMWT and enhanced, these T–F images were fed into the Sep-ResNet for the extraction of features and the classification of images to complete the recognition of RMS. At this point, the traditional neural networks will encounter some problems such as feature information loss, gradient vanishing, and gradient exploding as the network depth increases. It is hard to design a deeper network to extract the deep features of the images [21]. However, this paper uses the idea of residual learning, and introduces residual blocks and shortcut channels [22,23]. The classification network Sep-ResNet was designed, which solves problems of the loss of feature information, gradient vanishing, and gradient exploding. The Sep-ResNet can be designed to extract richer image features with greater depth. The Sep-ResNet structure was designed as shown in Figure 5.

**Figure 6:**
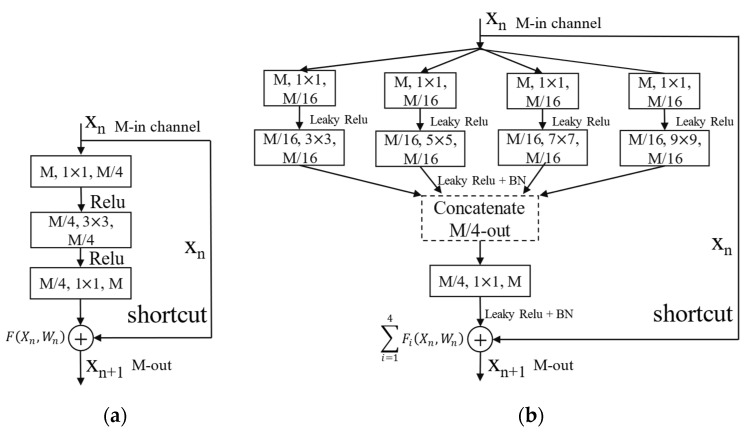
Comparison of the residual block. (**a**) is the original residual block of ResNet50 in [22]; (**b**) is the improved residual block in this paper. The residual block of (**b**) has multi-channel feature fusion and the multiple receptive fields.

In Figure 5, the Pre Conv uses three 3 × 3 convolution kernels to convolve the input image. The first one uses the convolution kernel step size s = 2 to conduct down sampling. The remaining two convolutions have the same receptive field as the original 7 × 7 convolution kernel, but the number of parameters is reduced by 45%. Furthermore, the features extracted by the smaller convolution kernel are more refined. Stage 1 includes two parts: the Down Sampling and the Residual Block. The Down Sampling part first adjusts the number of channels in Path A by using a 1 × 1 convolution kernel and then uses a kernel of s = 2 at the 3 × 3 convolution. Reference [22] uses s = 2 for convolution at the 1 × 1 convolution kernel, which will completely lose 50% of the information of the feature map. At Path B, the convolution operation with a size of 1 × 1 and s = 2 is also replaced with an average pooling with a size of 2 × 2 and s = 2. The above adjustment can ensure that the information of the feature map will not be lost when conducting the Down Sampling. The output of Down Sampling involves the width and height of the feature map being reduced by half, and the number of output channels is increased. Another part of the improved Residual Block is shown in Figure 6.

In Figure 6, the three parameters for convolution in the rectangle represent the input channel, kernel size, and output channel. The input channel of the residual block is M feature maps. The first layer of convolutional kernel size is 1 × 1 for convolution, and all of the four out channels are obtained M/16 feature maps. The obtained feature maps use the activation function of Leaky ReLu and conduct convolution with the kernel size of 3, 5, 7 and 9, respectively. The different sizes of convolutional kernels make it possible to obtain different receptive fields and extractions of the multi-scale features. The M/16 feature maps of four channels are stacked in a concatenated manner on the channel to obtain M/4 feature maps. This allows the obtaining of fused feature maps. The obtained feature maps use kernels size is 1 × 1 for convolution and mapping to obtain M feature maps. Finally, the obtained M feature maps and the original M feature maps before convolution are correspondingly added to each channel to obtain a residual block. This method has a larger receptive field than the original residual structure, and the extracted features are more abundant. Although the increase in residual block parameters is caused by increasing the receptive field of the kernels, there is no increase in the number of convolutions. The residual block only separates the channels instead of increasing the number of channels. The residual block output of Sep-ResNet is $X_{n+1}$, as follows:

(11) $$X_{n+1}=X_{n}+\overset{4}{\underset{i=1}{\sum }}F_{i}\left(X_{n},W_{n}\right)\;$$

where $F_{i}\left(X_{n},W_{n}\right)$ is the output of $X_{n}$ after convolution in the *i*-th channel. The improved residual block can extract the features of T–F images with multiple scales and multiple receptive fields. The Sep-ResNet extracted features are more abundant in low-SNR environments, which increases the recognition accuracy of the RMS. Stage 1 repeats the residual block 3 times, and the output of Stage 1 is the input of Stage 2. Stage 2 repeats the above residual block 4 times, and the output of Stage 2 is the input of Stage 3, and so on. The structure of Sep-ResNet has a total of 53 layers of the network, including 51 convolutional layers, one auto average-pool layer, and one fully connected layer. In the Batch Normalization (BN) layer, the data of a mini batch are normalized to uniformly distributed data with a mean of 0 and a variance of 1, which can better prevent the problems of overfitting and vanishing of gradients [24]. The activation function is Leaky ReLu: x=max{0.01x,x}.The initial learning rate (LR) of the training model is LR = 0.001, and the learning rate is adjusted to LR = LR * 0.5 every 20 epochs. As the number of iterations increases, the learning rate decreases, for a total of 100 epochs. The auto average pool makes the feature maps of any width and height become size = 1 × 1 feature maps and then maps them to seven types of radar modulation signals through a full connection. The final Softmax layer maps the output probabilities of each type to between 0 and 1.

The loss function in this experiment is cross-entropy, and label smoothing [25] is introduced to reduce the over-fitting of the model, as shown in Equation (12).

(12) $$y^{\prime }=\left(1-\varepsilon \right)\times y+\frac{\varepsilon }{K}\;$$

(13) $$loss=-\overset{n}{\underset{i=0}{\sum }}\left[y^{\prime }\times logp+\left(1-y^{\prime }\right)\times log(1-p)\right]$$

where y is the original label (named the hard label) and $y^{\prime }$ is the smoothing label (named the soft label), the allowable error rate ε is 0.1, the number of categories K is 7, p is the prediction result, and loss is the error between the prediction result and the given truth label. The hard label only has values of 0 and 1. If there is a label error, the model will learn the features of the image in the wrong direction, resulting in poor model generalization and easy overfitting. The soft label allows a certain error tolerance rate, which can alleviate the overfitting of the model. Considering that the signal characteristics are not obvious and are severely polluted by noise when the SNR is low, the signals can easily be misclassified in low-SNR environments. The addition of label smoothing also causes the model have a certain anti-noise ability, which alleviates the problem of the loss function of cross-entropy being easily overfitted. Finally, back propagation is used to update the weight parameters of each layer to complete the training of the RMS recognition model.

## 4. Experimental Results and Discussion

In this part, the experimental dataset and the results are given. According to the framework of Figure 1, the method processes radar signals to obtain T–F images, and the separable Sep-ResNet channels classify the enhanced T–F images. Our method accurately identified seven radar modulation signals in low-SNR environments of ≤−10 dB.

### 4.1. Experimental Dataset

The experimental environment used for the generation of the simulation signals and T–F images was MATLAB2018a. The deep learning framework for training and predicting model was Pytorch1.5.

The experimental data comprised the radar signal of seven modulation modes generated by simulation, namely NS, LFM, NLFM, 2FSK, 2PSK, 4FSK, and 4PSK. The frequency of the modulation signal was the normalized frequency, which was the signal frequency divided by the sampling frequency. Table 1 shows the specific modulation method, carrier frequency, bandwidth and Baker codes of RMS.

In Table 1, the simulated RMS parameters are the dynamic range [9]. To simulate the actual received signal, the modulated signal had the range of a certain parameter, and the noise was added. The SNR of radar modulation signals was −13, −10, −7, −4, −1, 2, 5 and 8 dB, respectively. An SNR point was taken every 3 dB, for a total of eight SNR points. A total of 400 T–F images were taken for each SNR point, and each radar modulation signal contained 3200 T–F images. There were a total of 22,400 T–F images in this dataset. Overall, 60% of the dataset was used as the training set—a total of 13,440 T–F images; 40% was used as the test set—a total of 8960 T–F images.

**Figure 7:**
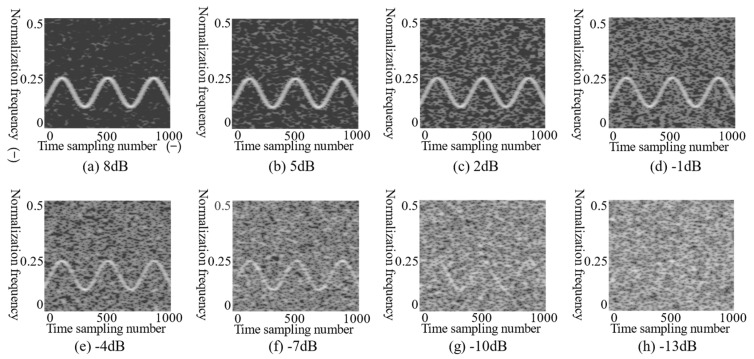
T–F images of the NLFM signal in different SNR from −13 dB to 8 dB in the training set. The images from (**a**)–(**h**) are NLFM grayscale T–F images under 8 dB, 5 dB, 2 dB, −1 dB, −4 dB, −7 dB, −10 dB and −13 dB noise, respectively.

The T–F images shown in Figure 7 could obtain the characteristics of the NLFM signal well, but the characteristics of the T–F images were gradually overwhelmed by noise as the SNR decreased. When the SNR was ≥−1 dB, the signal characteristics were clear in the T–F images. When the SNR was ≤−4 dB, the characteristics became vague. When the SNR was −10 dB, the noise seriously interfered with the signal characteristics. Furthermore, when the SRN was −13 dB, the characteristics were completely overwhelmed by noise.

### 4.2. Experimental Results

The T–F images of the RMS in the training set were fed into the CNN after denoising and enhancement to the train models for the recognition of the RMS.

#### 4.2.1. Verification of the Effectiveness of CMWT

**Figure 8:**
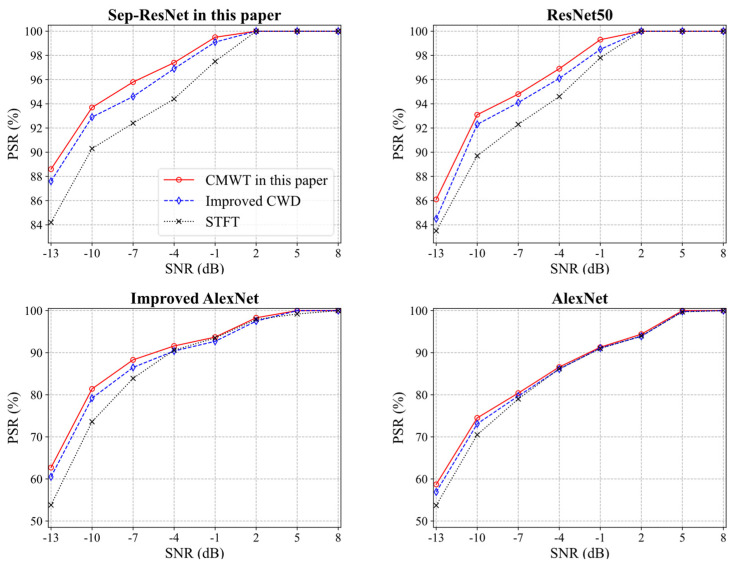
The PSR of three T–F analysis methods in different classification networks.

In this paper, the RMS used three T–F analysis methods, including STFT [15], CWD with an improved kernel function [14], and CMWT, to obtain the T–F images. The T–F images were fed into AlexNet, the improved AlexNet [12], and ResNet50 [22], and our Sep-ResNet for the classification of T–F images, which used an enhancement algorithm, is shown in Algorithm 1. The probability of the successful recognition (PSR) of different T–F analysis methods were compared, as shown in Figure 8:

In Figure 8, the red line is the CMWT in this paper, the blue line is the improved CWD, and the black line is the STFT. The T–F analysis method of CMWT had the highest PSR of the four networks, while the CWD method with an improved kernel function had the middle PSR, and the STFT method had the lowest PSR. Because CMWT had the advantage of containing wavelets with transformable scales, there were no cross-terms and phase information in the imaginary part. It showed anti-noise ability and effectively extracted the RMS characteristics. It was found that the improved T–F analysis method can improve the PSR of the RMS. For the four CNNs, the overall PSR values of the three T–F analysis methods of the CMWT, CWD, and STFT are shown in Table 2.

In Table 2, it can be seen that the T–F analysis method of the CMWT and the Sep-ResNet classification network had the highest PSR of the seven radar signals. The overall PSR of this method was 96.57%.

#### 4.2.2. Verification of the Effectiveness of Sep-ResNet

**Figure 9:**
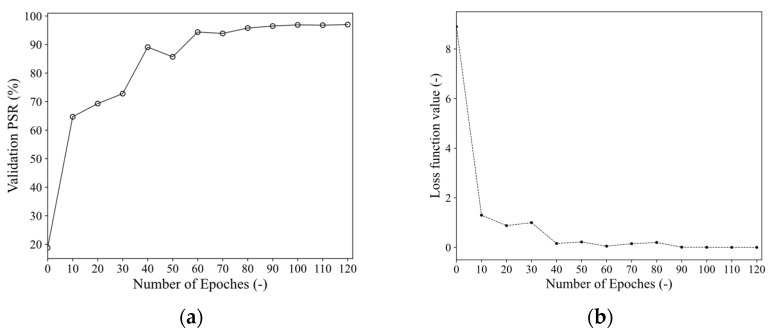
(**a**,**b**) are the curves of the validation accuracy and loss function value, respectively, obtained during the training of Sep-ResNet. The abscissa is the number of epochs. An epoch represents all samples in the training set being trained once. The ordinate of (**b**) is the specific loss function value under the current epoch.

To verify the effectiveness of the classification network of the proposed Sep-ResNet, the enhanced T–F images obtained via the CMWT were fed into the Sep-ResNet for training of the recognition model. The validation dataset was composed of 20% of the test dataset. Because the CMWT was able to better extract the RMS characteristics to obtain clear and distinguishable T–F images, the loss function successfully converged as the number of epochs increased, as shown Figure 9.

In the experiment, we conducted a classification comparison of AlexNet, ResNet50, VGGNet16, Inception-v3, and the backbone of U-Net models. The AlexNet structure was consistent with that reported in [12]. The residual structure of ResNet50 [22] is shown in Figure 6. VGGNet16 [26] increases the number of the output channels and uses max pooling to reduce the size of the feature map. VGGNet16 has a total of 16 convolutional layers, which is deeper than AlexNet. By decomposing large convolution filters such as 5 × 5 into two 3 × 3 filters, Inception-v3 [25] has improved performance on VGGNet. The parameters are reduced by 28%. Furthermore, Inception-v3 uses an asymmetric method to decompose the spatial convolution filter. The n × n size filter is decomposed into 1 × n and n × 1 filters to further reduce the parameters and improve performance. The main idea of U-Net is to use a feature pyramid network for feature fusion [27]. The U-Net described in this paper used a 3 × 3 size filter to convolve a T–F image of (1, 64, 64) size to obtain a feature map of (64, 32, 32) size, where the dimensions were the output channel, image width, and image height. These features were deconvolved again to obtain a feature map of size 128, 16, 16, the feature map was copied, and it was named as m1. Then, the convolution was continued to obtain the feature maps of size (256, 8, 8) and (512, 4, 4), which were named m2 and m3, respectively. m3 was upsampled to obtain feature maps of 512, 8, 8 size, and concatenated with m2 on the channel to obtain sizes of 768, 8, 8. Then, up-sampling allowed the feature maps of 768, 16, 16 size to be obtained, and these were added to m1 in order to obtain the feature maps of 896, 16, 16 size. Finally, four 3 × 3 size convolution filters and fully connected layers were mapped to seven classification nodes, corresponding to seven types of radar signals. The key to U-Net is the integration of deep and shallow features to better express image features.

**Figure 10:**
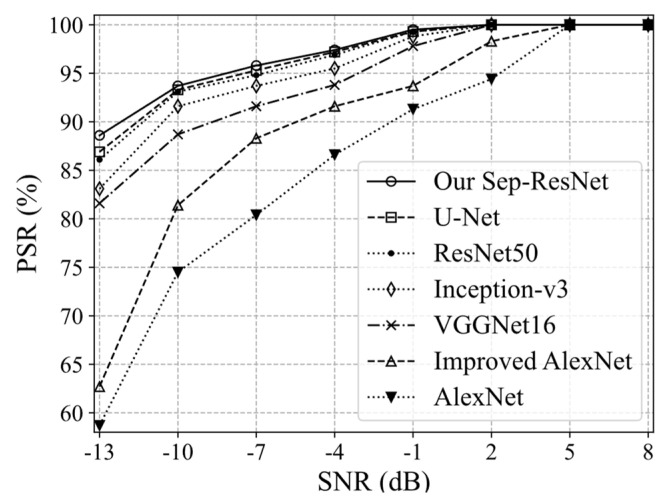
The PSR of seven CNNs.

During the experiment, the data processing method was consistent with the use of CMWT and the enhanced algorithm in Algorithm 1. The T–F images were fed to AlexNet, improved AlexNet, VGGNet16, Inception-v3, U-Net, ResNet50, and our Sep-ResNet, respectively. The PSR values of seven CNNs are shown in Figure 10.

The proposed Sep-ResNet had the highest PSR for the above seven radar modulation signals. Under SNR = −13 dB, the PSR of the Sep-ResNet was still 88.24%. The PSR of the AlexNet, improved AlexNet, VGGNet16, Inception-v3, ResNet50 and U-Net were 58.36%, 62.31%, 81.62%, 83.17, 85.92, and 86.81%, respectively. Because the residual network was able to design the deep enough network structure to extract T–F image features, we observed that Sep-ResNet has a multi-scale receptive field and multi-channel feature fusion, which can extract richer features of T–F images. It was found that Sep-ResNet has the best classification effect in low-SNR environments, improves the PSR, and has the anti-noise ability. Furthermore, the Sep-ResNet model showed a recognition rate of 100% for the above seven radar signals when the SNR was ≥2 dB.

**Figure 11:**
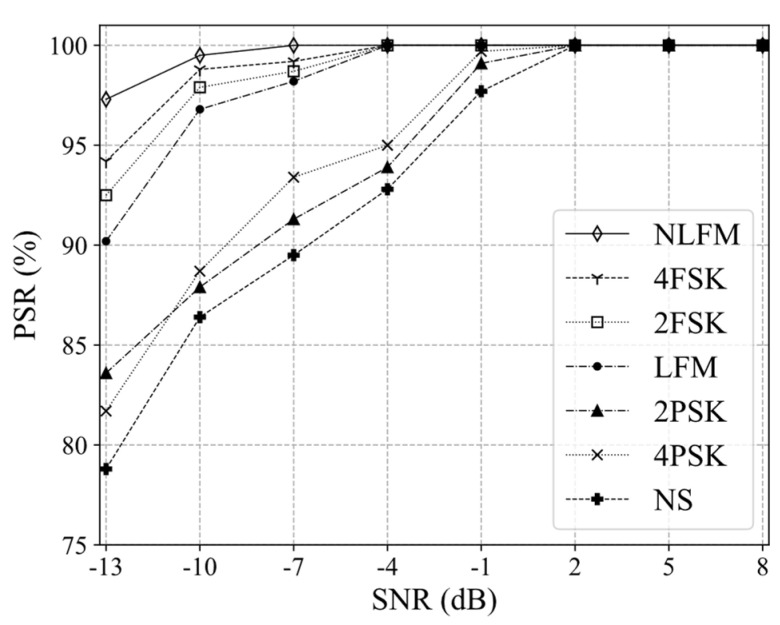
The PSR of seven RMSs in Sep-ResNet.

**Figure 12:**
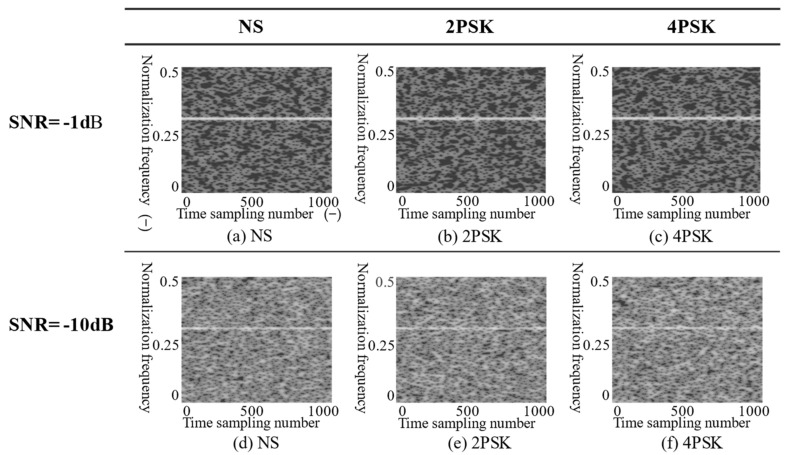
The T–F images of NS, 2PSK, and 4PSK when the SNR was −1 dB and −10 dB, respectively. The (**a**,**d**) are the grayscale T–F images of the signal NS under −1 dB and −10 dB noise, respectively. The (**b**,**e**) are the grayscale T–F images of the signal 2PSK under −1 dB and −10 dB noise, respectively. The (**c**,**f**) are the grayscale T–F images of the signal 4PSK under −1 dB and −10 dB noise, respectively.

Figure 11 shows the PSR of the Sep-ResNet model for seven radar modulation signals at different SNRs. Among the seven modulation signals, NLFM, 2FSK, 4FSK, and LFM had higher recognition rates. When the SNR was −13 dB, their average PSR was able to reach 93.45%, and when the SNR was ≥−4 dB, the PSR could reach 100%. The average PSR of the remaining signals—NS, 2PSK, and 4PSK—was 81.36% when the SNR was −13 dB. Their PSR was lower because the signal characteristics were relatively similar on the T–F images. Furthermore, with the decrease in SNR, the signal characteristics were overwhelmed by noise, resulting in low recognition accuracy (see Figure 12).

The input of the confusion matrix was 100 random enhanced T–F images from the test dataset by CMWT when the SNR was −4 dB. The output on the diagonal was the recognition recall rate of each radar modulation signal. The number of identification errors was divided on the diagonal. From Table 3, we can conclude that the Sep-ResNet model had identification errors for NS, 2PSK, and 4PSK. The PSR values of NS, 2PSK, and 4PSK were 92%, 94%, and 95%, respectively. These values could be identified with 100% accuracy for the remaining four types of signals—the NS, 2PSK, and 4FSK identify errors—because their T–F images were very similar. The strong noise interfered with the characteristics of the signal, which led to errors in the Sep-ResNet classification. The LFM, NLFM, 2FSK, and 4FSK had high recognition accuracy because the features of their T–F images were distinguishable. Therefore, it can be concluded that an effective T–F analysis method is very important for the identification of radar signals. The CMWT we proposed can obtain clear T–F images, and there are no cross-terms. Our Sep-ResNet also has good classification performance.

#### 4.2.3. Verification of the Effectiveness of T–F Image Enhancement

To verify the effectiveness of the proposed enhancement algorithm for T–F images, the T–F images obtained by CMWT were enhanced using the algorithm in Algorithm 1, which also shows the image-enhancement processing results.

**Figure 13:**
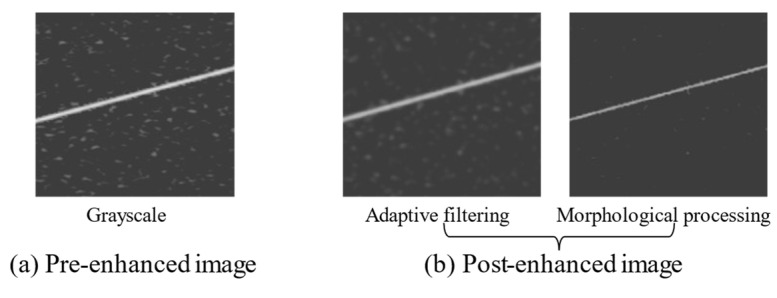
The LFM signal enhancement process at SNR = 8 dB. The (**a**) is the image before enhancement. The (**b**) is the image enhanced by the algorithm in this paper.

Figure 13b shows the grayscale T–F image of LFM signal after adaptive filtering and morphological processing. The noisy T–F images had better reductions in their noise interference, which was caused by their enhancement, and they retained the characteristics of the signal. The seven types of pre-enhanced and post-enhanced T–F images were fed into the Sep-ResNet model for recognition.

**Figure 14:**
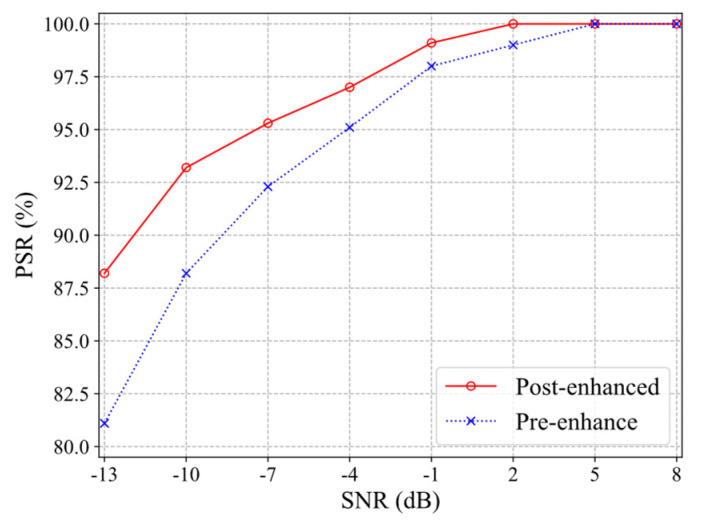
The PSR of the pre-enhancement and post-enhancement.

In Figure 14, it can be seen that after the T–F image enhancement algorithm was implemented, the overall PSR was improved by 2.35%, especially in the low-SNR −13, −10, −7, and −4 dB interval, by an average of 4.21%. When the SNR was −13 dB, the PSR was increased by 7.08%. The proposed enhancement algorithm could improve the PSR of radar-modulated signals in low-SNR environments. In general, a good image denoising and enhancement algorithm can reduce the interference of noise while retaining the characteristics of the signal, thereby improving the quality of T–F images and improving the system PSR of radar-modulated signals in low-SNR environments.

## 5. Conclusions

In response to the difficulty in identifying radar modulation signals in low-SNR environments, this paper proposed a method for combining the T–F analysis methods of CMWT and Sep-ResNet to intelligently identify radar modulation signals. In this paper, the T–F analysis of CMWT was used to extract the two-dimensional feature of the signal to obtain T–F images, and the images were enhanced through adaptive filtering and morphological processing. The enhanced T–F images were used as the input of Sep-ResNet for classification to intelligently and accurately achieve the recognition of radar-modulated signals in low-SNR environments. The experiments show that the T–F analysis of CMWT was better than the STFT and the improved CWD model. The classification performance of the proposed Sep-ResNet was better than AlexNet, the improved AlexNet, VGGNet16, Inception-v3, ResNet50, and the backbone of U-Net. Furthermore, the proposed enhancement algorithm was effective in filtering out the noise on T–F images. The method proposed successfully identified seven types of radar modulation signals (NS, LFM, NLFM, 2FSK, 2PSK, 4FSK, and 4PSK) in low-SNR environments. With SNR values ranging from −13 dB to 8 dB, the overall recognition rate was 96.57%, which was sufficient to effectively identify radar-modulated signals. Therefore, this method has the ability to resist noise interference, and can still maintain high PSR in low-SNR environments of ≤−10 dB, thereby avoiding the difficulty and instability involved in the manually identification of radar modulation signals.

## Figures and Tables

**Table 1:** The specific simulation parameters of RMS.

Signal Type	Parameter	Range
NS LFM	Carrier frequency $f_{c}$ $f_{c}$ Bandwidth △f	(180~230) MHz (180~230) MHz (40~60) MHz
NLFM	$f_{c}$ △f	(180~230) MHz (20~40) MHz
2PSK	$f_{c}$Barker codes Symbol width	(180~230) MHz Length = {7, 11, 13} 0.04 μs
2FSK	$f_{c1}$, $f_{c2}$ Barker codes Symbol width	(180~200), (280~300) MHz {7, 11, 13} 0.04 μs
4PSK	$f_{c}$ Baker codes Symbol width	(180~230) MHz {5, 7, 11, 13} 0.03 μs
4FSK	$f_{c1}$, $f_{c2}$ $f_{c3}$, $f_{c4}$ Baker codes Symbol width	(180~190), (210~220) MHz (240~250), (270~280) MHz {5, 7, 11, 13} 0.03 μs

Note: The pulse width for each type signal is 0.5 μs and sampling frequency is 2 GHZ.

**Table 2:** The overall PSR of RMS.

	STFT, %	CWD, %	Our CMWT, %
Our Sep-ResNet	94.59	96.05	96.57
ResNet50	94.53	95.43	95.89
Improved Alexnet	86.25	88.12	89.31
Alexnet	84.02	84.93	85.52

**Table 3:** The confusion matrix of Sep-ResNet model for signal recognition when the SNR was −4 dB.

Input	Output						
	NS	LFM	NLFM	2FSK	2PSK	4FSK	4PSK
NS	92	0	0	0	4	0	4
LFM	0	100	0	0	0	0	0
NLFM	0	0	100	0	0	0	0
2FSK	0	0	0	100	0	0	0
2PSK	3	2	0	0	94	0	1
4FSK	0	0	0	0	0	100	0
4PSK	3	0	0	0	2	0	95

## Data Availability

Not applicable.

## Abbreviations

The following abbreviations were used in this manuscript:

SNR	signal-to-noise ratio
T–F	time–frequency
CMWT	complex Morlet wavelet transform
Sep-ResNet	channel-separable residual network
PSR	probability of successful recognition
RMS	radar modulation signal
WT	wavelet transform
CWD	Choi–Williams distribution
STFT	short-time Fourier transform
